# 2020 Review and revision of the 2015 Darwin melioidosis treatment guideline; paradigm drift not shift

**DOI:** 10.1371/journal.pntd.0008659

**Published:** 2020-09-28

**Authors:** Richard P. Sullivan, Catherine S. Marshall, Nicholas M. Anstey, Linda Ward, Bart J. Currie

**Affiliations:** 1 Global and Tropical Health Division, Menzies School of Health Research, Charles Darwin University, Darwin, NT, Australia; 2 Department of Infectious Diseases, Royal Darwin Hospital, Darwin, NT, Australia; 3 Department of Infectious Diseases, Immunology and Sexual Health, St George Hospital, St George & Sutherland Clinical School, UNSW, Kogarah, NSW, Australia; University of Oxford, UNITED KINGDOM

## Abstract

**Background:**

Melioidosis therapy is divided into an intravenous intensive phase and an oral eradication phase. The Darwin melioidosis treatment guideline has evolved over two decades, with over 1150 consecutive patients with culture-confirmed melioidosis managed under the Darwin Prospective Melioidosis Study. The current guideline, published in 2015, has been associated with low rates of recrudescence, relapse and mortality, and together with the treatment trials in Thailand, forms the basis for consensus global guidelines.

We aimed to reassess the Darwin guideline and determine if any adjustments to the recommendations better reflect current practice in melioidosis therapy at Royal Darwin Hospital.

**Methodology/Principal findings:**

This retrospective cohort study reviews the characteristics, admission duration, duration of intravenous antibiotics, recrudescence, recurrence and mortality in all patients presenting with first episode culture-confirmed melioidosis in the tropical north of Australia’s Northern Territory from 1^st^ October 2012 until 1^st^ January 2017.

234 patients were available for analysis. 16 (6.8%) died during the intensive phase treatment and 6 (2.6%) did not have complete treatment at Royal Darwin Hospital, leaving 212 patients for analysis. Six (2.8%) patients had recrudescence during therapy and 10 (4.7%) had recurrent melioidosis (relapse or new infection) after completion of therapy. Persisting osteomyelitis requiring surgery was an important reason for recrudescence as was unrecognized osteomyelitis for relapse. For patients presenting with an antibiotic duration determining focus of pneumonia, durations of intravenous antibiotics were often prolonged beyond the current 2-week minimum treatment recommendation. Prolongation of therapy in pneumonia mostly occurred in patients presenting with multi-lobar disease or with concurrent blood culture positivity.

**Conclusions/Significance:**

The 2015 Darwin melioidosis guideline is working well with low rates of recrudescence, relapse and mortality. Based on the practice of the treating clinicians, the 2020 revision of the guideline has been adjusted to include a duration of a minimum of 3 weeks of intravenous antibiotics for those with concurrent bacteraemia and pneumonia involving only a single lobe and those with bilateral and unilateral multi-lobar pneumonias who do not have bacteraemia. We also extend to a minimum of 4 weeks intravenous therapy for those with concurrent bacteraemia and bilateral or unilateral multi-lobar pneumonia.

## Author summary

Melioidosis, caused by the Gram-negative bacterium *Burkholderia pseudomallei*, is an infectious disease with diverse clinical presentations including pneumonia, localised cutaneous lesion, bacteraemia without evident focus, septic arthritis and osteomyelitis, and severe sepsis with multiple organ abscesses. Therapy is prolonged, consisting of an intensive intravenous phase and an oral eradication phase. Guidelines from northern Australia have evolved and now recommend an often-longer duration of intravenous antibiotics than in prior recommendations. The Darwin melioidosis guideline, which was first published internationally in 2015 and described as a new treatment paradigm, has been associated with relatively low rates of recrudescence and relapse. We have reassessed use of this guideline and have again demonstrated low rates of recrudescence, relapse and mortality. There has been a tendency for treating clinicians to prolong intravenous phase therapy beyond the 2-week recommendation in those presenting with concurrent bacteraemia and pneumonia and in those with multi-lobar pneumonia. The 2015 guideline remains the standard for treatment recommendations for our region, but the 2020 revision now includes multi-lobar pneumonia as an indication for a minimum of 3 weeks intravenous treatment if bacteraemia is not present and 4 weeks if bacteraemia is present, while a minimum of 3 weeks is recommended for those with concurrent bacteraemia and pneumonia involving only a single lobe.

## Introduction

Melioidosis, caused by the Gram-negative bacterium *Burkholderia pseudomallei*, is an infectious disease with diverse clinical presentations including pneumonia, localised cutaneous lesion, bacteraemia without evident focus, septic arthritis and osteomyelitis, and severe sepsis with multiple organ abscesses [1–3]. It is endemic in southeast Asia and northern Australia and is increasingly recognised in other tropical and sub-tropical regions [4]. Modelling proposed that there were an estimated 165,000 human cases and 89,000 deaths worldwide per year [4]. Risk factors include diabetes mellitus, hazardous alcohol use, chronic lung disease, chronic renal disease and immunosuppression from disease or therapy, with only 20% of cases having no identifiable risk factor [1, 5].

*B pseudomallei* has broad intrinsic antimicrobial resistance and prolonged therapy is required for cure, comprising an intravenous intensive phase with ceftazidime or meropenem or imipenem, followed by an oral eradication phase of at least 12 weeks, usually with trimethoprim-sulfamethoxazole [1, 3, 6, 7]. The Darwin melioidosis treatment guideline has evolved over two decades, with over 1150 consecutive patients with culture-confirmed melioidosis managed under the Darwin Prospective Melioidosis Study [5, 8]. The Darwin melioidosis guideline as published in 2015 was described as a new treatment paradigm because of the focus on an often-longer duration of intravenous antibiotics than in prior recommendations, and was associated with relatively low rates of recrudescence and relapse [9]. The duration of intravenous therapy recommended is determined by the site, also known as the antibiotic duration determining focus, and severity of melioidosis [9]. Ceftazidime is used for most cases, with meropenem generally reserved for those with severe disease requiring admission to the Intensive Care Unit (ICU)[10, 11]. Oral trimethoprim-sulfamethoxazole is added during intensive phase therapy in cutaneous melioidosis, osteomyelitis or septic arthritis, central nervous system infection and with deep seated collections [9, 10]. The rationale for dual therapy is both the added tissue penetration and to potentially limit the emergence of resistance. Nevertheless, such dual therapy was not shown to improve mortality in patients with severe melioidosis in Thailand [12].

Together with guidelines evolved from three decades of treatment trials in Thailand, the Darwin melioidosis guideline, while reflecting the experience of a single centre, has formed the basis of consensus global guidelines under the auspices of the International Melioidosis Society. The Darwin melioidosis guideline [9] reflects the current treatment recommendation in resource rich countries [6], however it is recognised that prolonged intravenous therapy may not be possible or affordable in other regions [1, 6, 7]. In these regions, at least 10 days intravenous therapy is recommended [1]. The risk of melioidosis relapse was 9.7% in a large study in Thailand with choice and duration of oral eradication therapy being the most important determinants of relapse and duration of intravenous therapy not itself having an influence [13]. Nevertheless, relapse has become less common in our region despite poor adherence to oral eradication, and this has been attributed to the prolonged intravenous therapy phase [9, 14, 15].

The purpose of this study was to assess the actual duration of intravenous therapy and the outcomes of patients treated for melioidosis in northern Australia using the Darwin melioidosis treatment guideline, recognising that the guideline specifies for clinicians the minimum duration of intravenous therapy. Actual duration of intravenous antibiotics given, mortality, relapse and recrudescence were assessed. Changes to the 2015 Darwin melioidosis guideline [9] were then incorporated into the 2020 revised Darwin melioidosis treatment guideline, which is presented here.

## Methods

### Design

This was a retrospective analysis of data from medical records and also data collected from the Darwin Prospective Melioidosis Study [5, 8, 16]. This study is based on treatment at Royal Darwin Hospital, where all patients with melioidosis are managed by the Infectious Diseases Department, whose physicians all have extensive experience in treating melioidosis and work by consensus.

### Patients

All patients presenting with first episode culture-confirmed melioidosis in the tropical north of Australia’s Northern Territory between 1^st^ October 2012 and 1^st^ January 2017, as reported (16). These dates immediately follow the dates of the prior guidelines analysis as published in 2015 [9], while ensuring adequate follow up to detect relapse after 1^st^ January 2017, well exceeding the local median time to relapse of 8 months [5]. The medical records and outpatient letters of all patients were retrospectively reviewed. Duration of intravenous antibiotics, duration of hospital stay, duration of subsequent outpatient parenteral antibiotic therapy, recrudescence and recurrence (relapse or new infection) rates were collected with follow up until December 1st, 2019. Outpatient parenteral antibiotic therapy was either at usual home residence or at a self-care accommodation facility located near the hospital site for patients from remote communities. This admission was sometimes extended beyond the duration of parenteral antibiotics for other reasons e.g. monitoring for side effects from oral eradication therapy, rehabilitation or transport issues. Additional parameters such as site of infection, age, sex and risk factors were taken from data collected for the Darwin Prospective Melioidosis Study [5, 8]. If recrudescence or recurrence occurred, cases were analysed in more detail by two authors (BJC, RPS) to determine the factors linked to recurrence or recrudescence.

### Definitions

Recrudescence was defined as the development of clinical illness during the oral eradication phase with concurrent new culture of *B*. *pseudomallei* in a clinical specimen. Recurrence was defined as the development of clinical illness after the oral eradication phase, with new culture of *B*. *pseudomallei* in a clinical specimen. Recurrence was considered either relapse, in which the *B*. *pseudomallei* isolated was of the same genotype using multilocus sequence type as that from the original infection, or new infection, in which the new *B*. *pseudomallei* was a different genotype from that of the original infection.

The antibiotic duration determining focus determined the duration of intravenous antibiotics recommended and was ascertained as previously described from the primary diagnosis and/or concomitant collections [9]. If there were two or more foci in which the guideline recommended the same minimum duration of antibiotics, the primary diagnosis listed from the Darwin Prospective Melioidosis study was used [5, 8].

Duration of intravenous antibiotics was the duration actually received by the patient after the last culture positive drainage. Self-discharge was defined as a self-cessation of inpatient status for >24 hours or not attending for post dialysis intravenous antibiotics prior to the completion of clinician planned intensive phase.

### Data analysis

Continuous variables were expressed as median with interquartile ranges. Comparison between categorical variables was performed using the two-tailed Fisher exact test. Significance was set at p<0.05. 95% confidence intervals were calculated for proportions using the exact binomial method.

### Ethics

The study was approved by the Human Research Ethics Committee of the Northern Territory Department of Health and Menzies School of Health Research (HREC 02/38).

## Results

### Baseline characteristics

**Fig 1:**
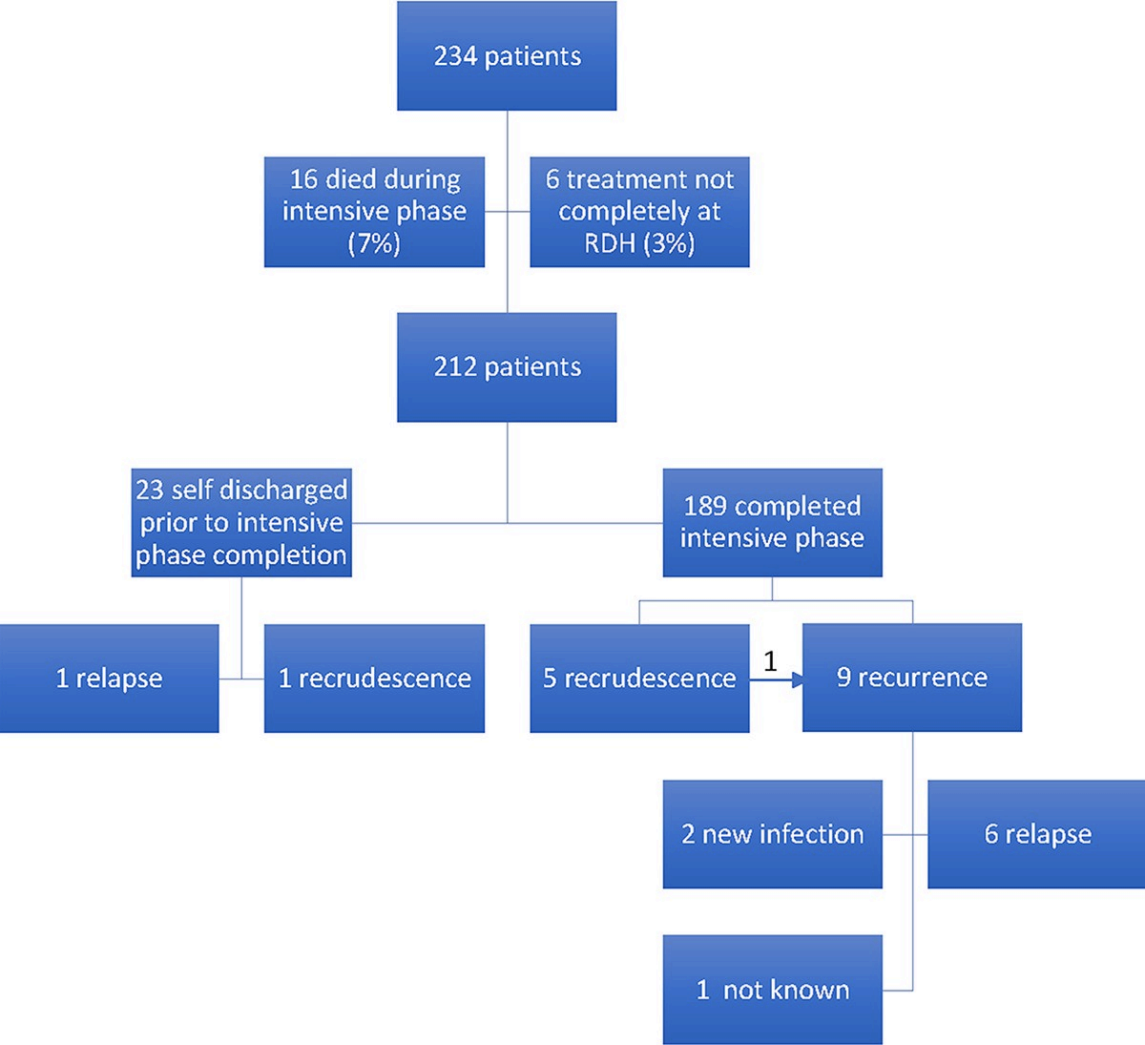
Patients treated for melioidosis in Northern Territory 1^st^ October 2012 to 1^st^ Jan 2017. (1 recrudescent case subsequently had a recurrence).

There were 234 patients presenting for the first time with culture-confirmed melioidosis between 1^st^ October 2012 and 1^st^ January 2017 [16]. Of these, 16 (6.8%) died during the intensive phase treatment and 6 (2.6%) did not have complete treatment at Royal Darwin Hospital (See Fig 1). Baseline characteristics of the remaining 212 patients, are given in Table 1 [16], with 97 (45.8%) having pneumonia as their antibiotic duration determining focus.

**Table 1:** Baseline Characteristics of 212 included patients [16].

Characteristic	Number (%) except where indicated
Male	125 (59.0)
Age (median)	51 years (IQR 39–60)
Any risk factor - Diabetes - Hazardous alcohol use - Chronic renal disease - Chronic lung disease - Malignancy - Immunosuppressive therapy - Rheumatic heart disease or congestive cardiac failure - Kava use - Other	193 (91.0) 105 (49.5) 98 (46.2) 31 (14.6) 61 (28.8) 20 (9.4) 21 (9.9) 20 (9.4) 4 (1.9) 12 (5.7)
Antibiotic duration determining focus - Skin - Bacteremia no focus - Pneumonia - Deep-seated collection - Osteomyelitis - Central nervous system infection - Arterial infection	28 (13.2) 13 (6.1) 97 (45.8) 53(25.0) 18 (8.5) 2 (1.0) 1 (0.5)
Duration of intravenous antibiotics post last culture positive drain (median)	28 (IQR 15–32) days
ICU admission	46 (21.7)
Duration of hospital admission (median)	18 (IQR 8–36) days
Outpatient parenteral antibiotic therapy admission	154 (72.6)
Duration of outpatient parenteral antibiotic therapy admission (median)	20 (IQR 13–26) days
Readmission during outpatient parenteral antibiotic therapy	32/154 (20.8%)
Self-discharge	23 (10.8)

### Recrudescence and relapse

Six (2.8%) of the 212 patients had recrudescence and 10 (4.7%) had recurrence (of which 1 had previously been a recrudescence and 7 were relapses) (See Fig 1). Table 2 shows the details of these recrudescence and recurrence cases, including the sequence types for each relapse. 7 (3.3%) patients died during oral eradication therapy, all from causes unrelated to melioidosis.

**Table 2:** Recrudescence and recurrence.

(DPMS ID)	Antibiotic Duration Determining Focus	Total IV duration (days) ††	Time from admission to recrudescence or recurrence (days)	Reason for recrudescence or recurrence	Multilocus Sequence Type
**Recrudescence**					
1(895)	Osteomyelitis	56	175	Osteomyelitis	.
2(989)	Pneumonia (4w)	34	89	Poor adherence to oral eradication therapy	.
3*(856)	Osteomyelitis**	13	52	Osteomyelitis	.
4 (863)	Deep seated collection	22	65	Self-discharge	.
5 (877)	Pneumonia (4w)	32	75	Incomplete oral eradication therapy	.
6 (984)	Osteomyelitis	43	119	Osteomyelitis	.
**Recurrence**					
1 (930)	Pneumonia (2w)	27	1092	New infection	Reinfection
2(1002)	Pneumonia (4w)	26	177	Unrecognized mycotic aneurysm	36
3* (856)	Osteomyelitis	13	589	Osteomyelitis	553
4 (831)	Deep seated collection†	20	134	Self-discharge	109
5 (852)	Deep seated collection†	30	249	Incomplete oral eradication therapy	1344
6 (912)	Deep seated collection	29	1318	Unrecognized osteomyelitis	1377
7(954)	Deep seated collection†	28	144	Incomplete oral eradication therapy	36
8 (971)	Deep seated collection	28	548	Unrecognized osteomyelitis	Paired sample not available
9 (979)	Deep seated collection†	28	202	Incomplete oral eradication therapy	483
10 (1015)	Pneumonia	28	1103	New infection	Reinfection

*Same individual.

**Had three episodes of recrudescence

†Apparent adequate initial drainage was achieved.

†† Duration of IV antibiotics after last drainage

In the 189 completing intensive therapy, overall 13 (6.9%) had either recrudescence or recurrence. 2/23 (8.7%) of those who self-discharged before completing intravenous therapy had either recrudescence or recurrence, with 14/23 (60.1%) having pneumonia as their antibiotic duration determining focus. Deep seated collections were the antibiotic duration determining foci of infection in both of the self-discharged patients who failed therapy.

### Duration of intravenous antibiotics

**Fig 2:**
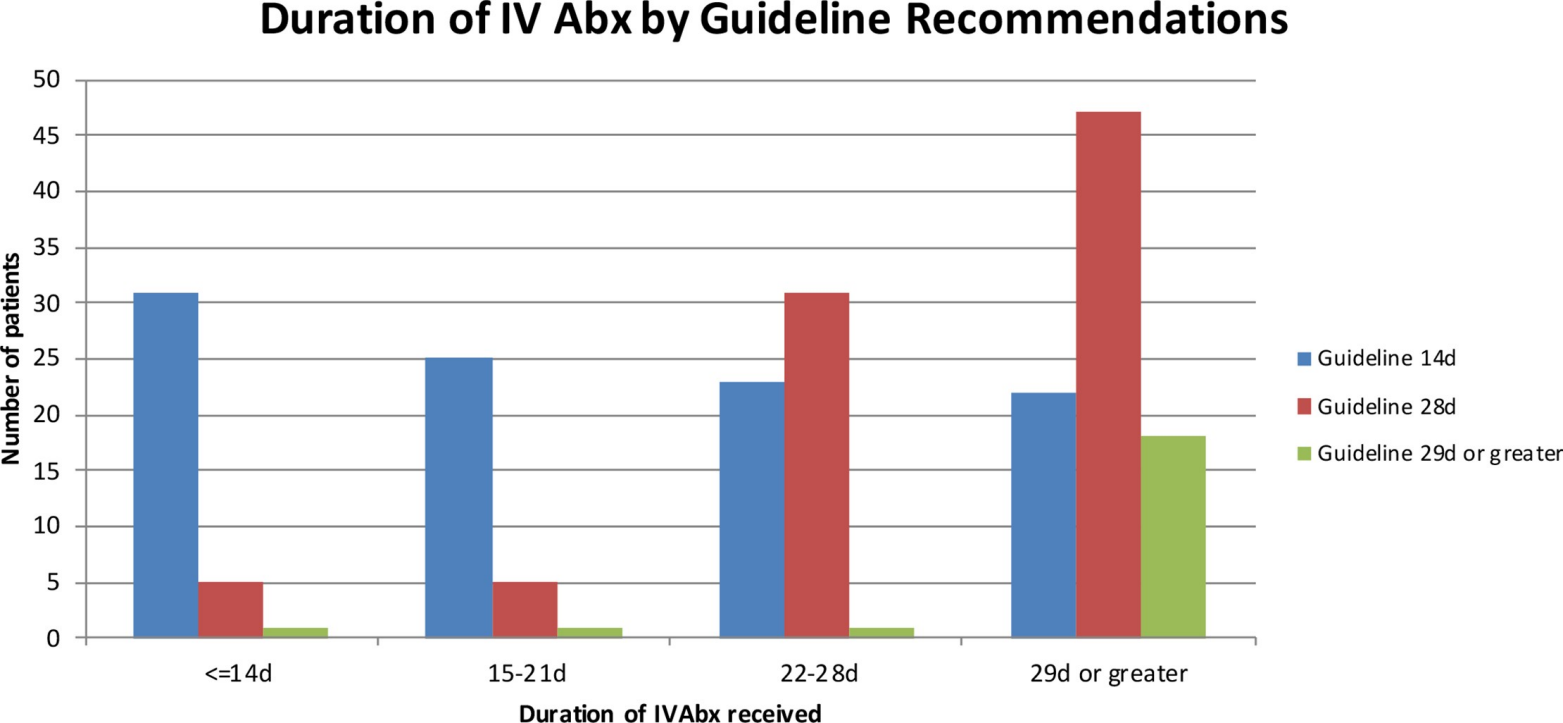
Duration of Intravenous Antibiotics received, broken down by Guideline Recommendations (n = 211, 1 patient with *B pseudomallei* isolated from a skin wound was not treated and excluded).

The actual durations of intravenous antibiotics given to patients are shown in Fig 2, categorised by their guideline-recommended duration group. There was a tendency to extend intravenous therapy beyond the guideline recommendation, which was seen predominantly in the group where the antibiotic duration determining focus was pneumonia.

**Fig 3:**
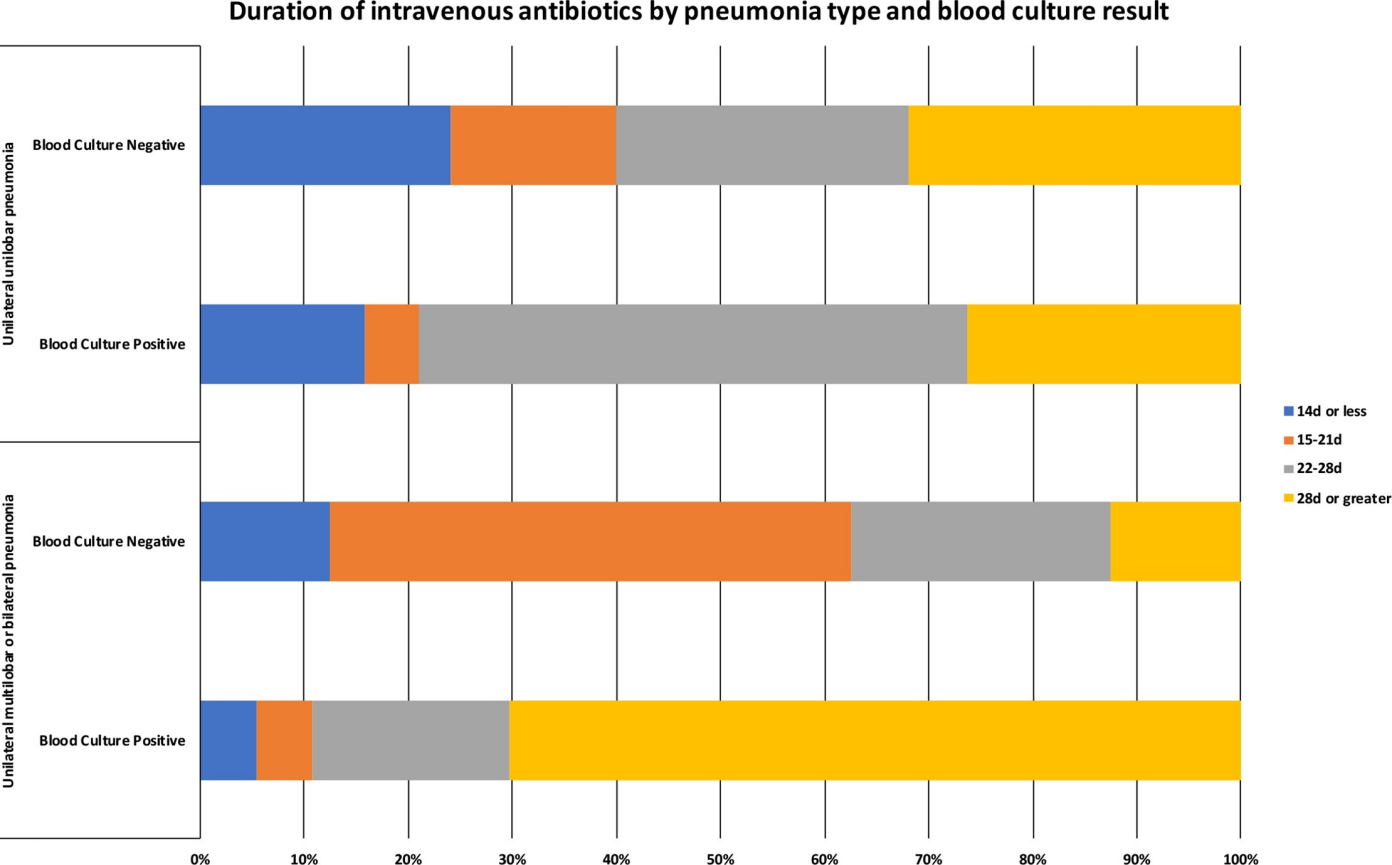
Duration of intravenous antibiotics in patients with a primary diagnosis of pneumonia by pneumonia type and blood culture result (n = 97).

The duration of antibiotics given to patients with an antibiotic duration determining focus of pneumonia are given in Table 3 and Fig 3, while Table 4 excludes those who self-discharged. Of those self-discharging with pneumonia (n = 14), none of whom had recurrent disease, the median duration of IV antibiotics was 15 (range 1–47) days.

**Table 3:** Duration of intravenous antibiotics in patients with primary diagnosis of pneumonia.

Current Guideline Recommendations	Chest x-ray changes and blood culture results	Duration of intravenous antibiotics received (n, %)				Total
		**< = 14 d**	**15–21d**	**22–28d**	**>28d**	
Current recommendation 2 weeks	Unilateral unilobar chest x-ray changes blood culture negative	6 (27.3)	4 (18.2)	6 (27.3)	6 (27.3)	22 (100)
Current recommendation 2 weeks	Unilateral unilobar chest x-ray changes, blood culture positive	3 (20.0)	1 (6.7)	7 (46.7)	4 (26.7)	15 (100)
Current recommendation 2 weeks	Unilateral multilobar or bilateral chest x-ray changes blood culture negative	1 (7.7)	8 (61.5)	4 (30.8)	0 (0)	13 (100)
Current recommendation 2 weeks	Unilateral multilobar or bilateral chest x-ray changes blood culture positive	1 (8.3)	1 (8.3)	3 (25.0)	7 (58.3)	12 (100)
Current recommendations 4 weeks	-	2 (5.7)	1 (2.6)	8 (22.9)	24 (68.6)	35 (100)
Total		13 (13.4)	15 (15.5)	28 (28.9)	41 (42.3)	97 (100)

**Table 4:** Duration of intravenous antibiotics in patients with primary diagnosis of pneumonia (excluding self-discharges).

Current Guideline Recommendations	Chest x-ray changes and blood culture results	Duration of intravenous antibiotics received (n, %)				Total
		**< = 14 d**	**15–21d**	**22–28d**	**>28d**	
Current recommendation 2 weeks	Unilateral unilobar chest x-ray changes blood culture negative	4 (20.0)	4 (20.0)	6 (30.0)	6 (30.0)	20 (100)
Current recommendation 2 weeks	Unilateral unilobar chest x-ray changes, blood culture positive	0 (0)	1 (8.3)	7 (58.3)	4 (33.3)	12 (100)
Current recommendation 2 weeks	Unilateral multilobar or bilateral chest x-ray changes blood culture negative	1 (11.1)	5 (55.6)	2 (22.2)	0	9 (100)
Current recommendation 2 weeks	Unilateral multilobar or bilateral chest x-ray changes blood culture positive	1 (9.1)	1 (9.1)	3 (27.3)	6 (54.5)	11 (100)
Current recommendations 4 weeks	-	1 (3.2)	0 (0)	7 (22.6)	23 (74.2)	31 (100)
Total		7 (8.4)	11 (13.3)	25 (30.1)	39 (47.0)	83 (100)

In examining the pneumonia groups in which 2 weeks is currently recommended, those with bilateral or unilateral multi lobar pneumonia received < = 14 days (2/25; 8%, 95%CI 1.0–26.0) less frequently compared to those with unilateral unilobar pneumonia (9/37; 24%, 95% CI 11.8–41.2), however, this was not statistically significant (Fisher’s exact test, p = 0.174). Additionally, those with unilateral unilobar pneumonia who were blood culture positive received < = 14 days (3/15; 20%, 95% CI 4.3–48.1) less frequently than those who were blood culture negative (6/22, 27.2%, 95% CI 10.7–50.2), although not statistically significant (Fisher’s exact test, p = 0.711). We compared the frequency of <21 days of therapy between those already recommended 4 weeks by the current guideline (3/35, 8.6%, 95%CI 1.8–23.1) and those recommended 2 weeks by the current guideline: there were statistically significantly more receiving <21 days in the unilobar, unilateral pneumonia blood culture negative group (10/22, 45.5%, 95%CI 24.4–67.8) and the multilobar blood culture negative group (9/13, 69.2%, 95%CI 38.6–90.9), but there was no significant difference with the unilateral unilobar blood culture positive group (4/15, 26.7%, 95%CI 7.8–55.1) and multilobar pneumonia blood culture positive group (2/12, 1.7%, 95%CI 2.1–48.4). These associations held when self-discharges were excluded. In those 35 patients currently recommended 4 weeks of IV therapy, there were only 3 who were unilateral unilobar and blood culture negative. The reason for the extension to 4 weeks in these 3 patients was the presence of mediastinal lymphadenopathy as seen on chest CT scan.

In the 62 patients in whom the antibiotic duration determining focus was pneumonia and current guideline recommendations were a minimum of 2 weeks there was no recrudescence. In the 35 patients in whom the antibiotic duration determining focus was pneumonia and current recommendations were a minimum of 4 weeks there were two with recrudescence despite >4 weeks of intravenous therapy. These were both explained by poor adherence to oral eradication therapy. There was also one relapse in one patient who had 26 days of intravenous therapy, attributed to an unrecognized deep focus. All episodes of recrudescence and relapse in this group had concomitant blood culture positivity during their first admission.

In the 13 patients with bacteraemia no focus as the antibiotic duration determining focus, clinicians also extended intravenous antibiotics beyond the recommended minimum 14 days in 9 patients. There were no recrudescence and no relapses in this group.

It was notable that in the four patients in whom relapsed melioidosis was attributed to incomplete oral eradication therapy (n = 3) or self-discharge (n = 1) (Table 2), all had had deep seated collections with apparently adequate drainage, and with three of the four having the guideline-appropriate duration of initial intravenous antibiotics of ≥4 weeks.

## Discussion

The patients in this melioidosis cohort had similar demographics, high risk factor rates, clinical presentations and antibiotic duration determining foci as in the prior cohort [9]. Outcomes confirmed that the 2015 Darwin melioidosis treatment guideline results in low rates of recrudescence and relapse, with those experiencing such events mostly explainable (Table 2). Some relapses were due to unrecognised foci or osteomyelitis while recrudescence occurred most often in the setting of osteomyelitis, similar to our previous series [9].

The recrudescence and relapse events highlight the importance of debridement and drainage in managing melioidosis osteomyelitis, septic arthritis and deep-seated abscess. Operative debridement is associated with lower rates of representation in patients with septic arthritis and osteomyelitis, and these patients often require multiple procedures [1, 17]. In addition, large deep abscess, such as prostatic abscesses require drainage for cure [1, 8, 18]. There was one case in which there was unrecognised arterial infection causing relapse, and in these cases urgent surgery and insertion of grafts are often needed, followed by long term suppressive oral therapy [1, 10, 19].

Nevertheless, failure to complete the oral eradication therapy phase was implicated in some recrudescence and relapse cases, notably despite substantial durations of initial intravenous therapy (Table 2). Despite the recognised poor adherence to and completion of the oral phase in our patients [9], this cautions against abandoning the oral eradication phase especially in those with more extensive bacterial burden such as deep-seated collections.

The rate of ICU admission is similar to our previous cohorts [5, 8], and improved ICU care has been postulated as one of the main drivers for the decreased mortality we have seen in our region over time [20]. The mortality rate of 6.8% seen in this cohort is lower than in our previous reports [5, 8].

After initial stabilisation and management in hospital the majority of patients were treated with outpatient parental antibiotic therapy. This therapy is given via a Peripheral Inserted Central Catheter (PICC) with an elastomeric infuser device and has been previously shown to be safe and effective in an out of hospital program [21]. The readmission rate from outpatient parenteral antibiotic therapy in our patient population was 20.8% (32/154). This highlights the complexities and difficulties in treating melioidosis and the need to closely monitor for new symptoms and signs in these settings. In a region such as northern Australia, where there are very isolated communities, the attachment of self-care accommodation facilities near the hospital to facilitate outpatient treatment in individuals from remote communities has allowed close monitoring, quick review and readmission as required. There were PICC related complications noted in two patients (one thrombosis and one infection), one of whom had been treated with outpatient parenteral antibiotic therapy.

While it is noted that the 2015 Darwin melioidosis treatment guideline defines the minimum duration of intravenous therapy, our data show that frequently the consensus clinical decision was to prolong the intravenous therapy beyond the minimum recommended for the patient’s antibiotic duration determining focus. The duration of intravenous antibiotics was extended beyond minimum guideline recommendation most commonly in patients who were presenting with an antibiotic duration determining focus of pneumonia. Analysis by other clinical parameters showed that this prolongation was frequently in patients who presented with bilateral pneumonia or those with unilateral multilobar changes, who in the 2015 guideline were recommended to have a minimum of 2 weeks of intravenous therapy. Clinician concerns about disease extent and a subjective impression of slower recovery in this group of patients resulted in the tendency to prolong therapy. Similar concerns resulted in clinician prolongation of intravenous therapy in patients with pneumonia and bacteraemia. Indeed, large studies from Thailand have shown relapse to be more common in patients with severe disease compared with those with localised melioidosis [22]. Other causes for clinician prolongation of intravenous therapy may include intolerance to first line eradication therapy with trimethoprim-sulfamethoxazole, common in these at-risk groups with comorbidities [16], and a perceived need to extend induction therapy when there is use of second-line oral eradication therapy with doxycycline. Of the 61 patients who experienced oral therapy side effects [16], the median duration of intravenous therapy was 28 days (IQR 15–42). In those who did not experience oral therapy side effects the median duration of intravenous therapy was also 28 days (IQR 15–30).

We postulate that the presence of multi-lobar pneumonia and/or concurrent bacteraemia and pneumonia are markers for more extensive and severe disease in our region. These groups reflected the majority of those in the current 2-week minimum intravenous therapy recommendation where intravenous therapy was prolonged by the clinicians beyond 2 weeks. Indeed, all three patients who had an antibiotic duration determining focus of pneumonia and experienced recrudescence or relapse, were blood culture positive.

We believe the ongoing changes in clinician practice with regard to the duration of intravenous therapy of patients with melioidosis pneumonia at Royal Darwin Hospital has contributed to the continued low rates of recrudescence and relapse in our region and most importantly the continuing downward trend in mortality.

To reflect this evolving “best practice” of the Darwin infectious diseases physicians, consensus was reached among the clinicians to formally incorporate changes into a revised guideline. The 2020 revision of the Darwin melioidosis treatment guideline now includes a duration of a minimum of 3 weeks of intravenous antibiotics for those with concurrent bacteraemia and pneumonia involving only a single lobe and those with bilateral and unilateral multi-lobar pneumonias who do not have bacteraemia. Minimum intravenous therapy is extended further to 4 weeks in those with bilateral and unilateral multi-lobar pneumonias who have bacteraemia. (See Table 5).

**Table 5:** 2020 Revised Darwin melioidosis guideline.

Antibiotic Duration Determining Focus	Minimum intensive phase duration (weeks)^a^	Eradication phase duration (months) ^f^
Skin abscess	2	3
Bacteraemia with no focus	2	3
Unilateral unilobar pneumonia without lymphadenopathy^b^, ICU admission, and with negative blood cultures	2	3
Multilobar unilateral or bilateral pneumonia without lymphadenopathy^b^, ICU admission and with negative blood cultures OR Unilateral unilobar pneumonia without lymphadenopathy^b^, ICU admission, but with positive blood cultures	3	3
Pneumonia with either lymphadenopathy^b^ or ICU admission OR Multilobar unilateral or bilateral pneumonia with positive blood cultures	4	3
Deep-seated collection^c^	4^d^	3
Osteomyelitis	6	6
Central nervous system infection	8	6
Arterial infection^e^	8^d^	6 ^g^

^a^ Clinical judgement to guide prolongation of intensive phase if improvement is slow or if blood cultures remain positive at 7 days

^b^ Defined as enlargement of any hilar or mediastinal lymph node to greater than 10 mm diameter

^c^ Defined as abscess anywhere other than skin, lungs, bone, CNS or vasculature. Septic arthritis is considered a deep-seated collection

^d^ Intensive phase duration is timed from the date of the most recent drainage or resection where culture of the drainage specimen or resected material grew *B*. *pseudomallei* or where no specimen was sent for culture; clock is not reset if specimen is culture-negative

^e^ Most commonly presenting as mycotic aneurysm

^f^ If concurrent oral therapy is not indicated in the intensive phase, oral eradication therapy to commence at the start of the final week of planned intensive intravenous therapy, with the timing of eradication duration commencing from the day after last intravenous therapy.

^g^ Life-long suppressive antibiotic therapy may be required following vascular prosthetic surgery.

While most data were collected prospectively as part of the Darwin Prospective Melioidosis Study, one limitation of this study is that the duration of antibiotic therapy and admission were collected retrospectively. In addition, we have not collected specific data for adherence to oral eradication therapy, but note that the prior analysis and ongoing clinical experience show adherence to be poor in a substantial proportion of patients [9]. Finally, although the revised guidelines reflect the consensus clinical experience, we do not have specific data on fever clearance or other markers of progress to support the impression of a slower clinical response in those for whom the revision now recommends a longer duration of antibiotic therapy.

In conclusion, the 2015 Darwin melioidosis guideline [9] has been modified in 2020 to reflect clinician practice of prolonging intravenous therapy beyond 2 weeks in patients with concurrent bacteraemia and pneumonia and those with unilateral multilobar or bilateral pneumonia. We acknowledge however that there are many regions endemic for melioidosis where such prolonged hospitalisation and intravenous therapy is often not possible or affordable [6, 7].
